# Effect of potassium-competitive acid blockers on human gut microbiota: a systematic review and meta-analysis

**DOI:** 10.3389/fphar.2023.1269125

**Published:** 2023-12-13

**Authors:** Meng-Ling Ouyang, Shu-Peng Zou, Qian Cheng, Xuan Shi, Ya-Zheng Zhao, Ming-Hui Sun

**Affiliations:** Department of Pharmacy, Tongji Hospital, Tongji Medical College, Huazhong University of Science and Technology, Wuhan, China

**Keywords:** vonoprazan, gut microbiota, *Helicobacter pylori*, alpha diversity, meta-analysis

## Abstract

**Background:** Vonoprazan has been reported to exert more potent and long-lasting gastric acid inhibition than proton pump inhibitors, potentially leading to a greater impact on the gut microbiota. This study aimed to clarify changes in microbial diversity and bacterial composition after VPZ treatments.

**Methods:** We searched from PubMed, Embase, WOS, Scopus, Cochrane Library, and *ClinicalTrials.gov* (all years up to May 2023). The primary outcomes were alpha and beta diversity, as well as differences in gut microbiota composition between before and after VPZ treatments. We performed a meta-analysis to uncover the potential changes in human gut microbiota among VPZ users by pooled mean difference (MD) with a 95% confidence interval (CI). The risk of bias was assessed using the ROBINS-I tool.

**Results:** A total of 12 studies were included to compare differences before and after VPZ treatments. Compared with baseline, alpha diversity was significantly reduced after VPZ treatments and gradually returned to baseline with longer follow-up. At the phylum level, there was a decrease in the relative abundance of *Firmicutes* and *Actinobacteria*, while *Bacteroidetes* increased compared with baseline. At the genus level, we found a significant decrease in the relative abundance of *Coprococcus* and *Bifidobacterium* and a significant increase in the relative abundance of *Bacteroides* compared with those before treatment. In subgroup analyses according to country and participants, we found differences in microbial changes after VPZ treatments.

**Conclusion:** Vonoprazan can affect the changes of gut microbiota, which may be potentially associated with its strong ability of acid inhibition. However, due to the large heterogeneity, further studies are required to validate these findings.

**Systematic Review Registration:** https://www.crd.york.ac.uk/prospero/, identifier CRD42023412265.

## Introduction

Vonoprazan (VPZ), as a novel potassium competitive acid blocker (P-CAB), utilizes a novel acid inhibition mechanism and overcomes many limitations of traditional proton pump inhibitors (PPIs) (Abdel-Aziz et al., 2021). Because of binding to both resting and activated H^+^/K^+^-ATPase and dissociating slowly, the P-CABs show rapid onset and sustained acid inhibition (Sakurai et al., 2015; Scott et al., 2015). Also, food has minimal effect on intestinal absorption of VPZ (Echizen, 2016). In addition, it is mainly metabolized by CYP3A4 and CYP2B6 enzymes, and less affected by CYP2C19 gene polymorphism, with little individual variation between patients (Inatomi et al., 2016; Graham and Dore, 2018; Wang et al., 2022). At present, it has shown good efficacy and safety in the treatment of gastroesophageal reflux, peptic ulcer, *Helicobacter pylori* (*H.pylori*) infection and other acid-related diseases (Kamada et al., 2021; Rokkas et al., 2021; Iwakiri et al., 2022). However, Taketo et al. reported that long-term use of VPZ may be more likely to affect crucial microbiota involved in preventing intestinal infections than traditional PPIs, thereby increasing the risk of intestinal microbiota infections (Otsuka et al., 2017).

The gut microbiota has many significant functions in the human body, including nutrient metabolism, barrier protection, immune regulation, influence of brain function and behavior, and regulation of bone density. Hence, the gut microbiota plays a crucial role in maintaining normal human physiology and health (Singh et al., 2017; Gomaa, 2020). Increasing evidence has shown that intestinal microbiota imbalance is related to obesity, diabetes, inflammatory bowel disease, colorectal cancer and other diseases (Sharma and Tripathi, 2019; Kim and Lee, 2021; Geng et al., 2022; Shastri et al., 2022). Previous studies have shown that VPZ and PPIs are significantly associated with the development of *Clostridioides difficile* infection (CDI) (Ishida et al., 2022). Acid suppression drugs decrease intragastric pH and weaken the protective effect of the gastric acid barrier, leading to the imbalance of gut microbiota. This promotes the proliferation of *Clostridium difficile* and the release of toxins, eventually leading to the occurrence of CDI (Watanabe et al., 2021). Furthermore, long-term PPIs treatments lead to intestinal dysbiosis and increase the risk of functional dyspepsia, spontaneous bacterial peritonitis, *Salmonella* and *Campylobacter* infections (Singh et al., 2018; Perry et al., 2020).

Gastric acid creates a low acidic environment that affects the growth of most pathogenic bacteria, providing nonspecific protection to the digestive tract (Tsuda et al., 2015). Because of the potent and sustained acid inhibition of VPZ, it is possible that VPZ may have a greater impact on the gut microbiota than PPIs(Otsuka et al., 2017). Therefore, it is important to understand the relationship between gut microbiota and VPZ treatment. This systematic review aims to compare the differences in microbial diversity and bacterial composition between VPZ and non-VPZ treatments. We also discuss the potential relationship between changes in gut microbiota and VPZ-related adverse effects.

## Materials and methods

### Search strategy and data sources

This systematic review was pre-registered at PROSPERO (CRD42023412265) and according to the guidelines of Preferred Reporting Items for Systematic Reviews and Meta-Analyses (PRISMA) (Checkist, Supplementary Table S1) (Page et al., 2021).

We searched PubMed, Embase, Web of Science (WOS), Scopus, Cochrane Library, and *ClinicalTrials.gov* (all years up to May 2023) and used the following keywords: potassium-competitive acid blockers, vonoprazan, tegoprazan, revaprazan, keverprazan, gut microbiota, and their types. The detailed search strategy for each database is shown in Supplementary Table S2. In addition, other data sources were considered, such as the possible references cited in included studies and relevant review articles.

### Study selection

We formulated the inclusion and exclusion criteria before database searching. Articles that met the following criteria were included: 1) participant: normal or diseased people; 2) intervention: after vonoprazan treatment; 3) comparator: before vonoprazan treatment; 4) outcome: gut microbial diversity and composition; 5) study: randomized controlled trial or observational study.

Studies were excluded according to the following criteria: 1) lack of data on gut microbiota; 2) cell and animal studies; 3) The gut microbiome was measured in samples other than feces; 4) gut microbiome detection without the use of high-throughput sequencing technology.

Two researchers (Shi X and Chen Q) independently screened titles and abstracts, as well as reviewed full text based on the inclusion and exclusion criteria from the databases. When a disagreement appeared, another investigator participated with others in the discussion of the disputed study until a consensus was reached.

### Data extraction and outcomes

Two researchers (Ouyang ML and Zou SP) designed the data extraction form and extracted the data independently. When necessary, the corresponding authors of the included studies were contacted for the needed information. For each included study, the following information were extracted: study ID (first author and publication year), country, study design, concomitant antibiotics, study population, sample size, DNA extraction methods, sequencing platform, outcomes assessed, microbiome composition, and diversity results before-after medication. For studies that had not shown the corresponding results, the Engauge Digitizer v.11.1 software was used to extract the required data from charts and graphs in the literature.

The primary outcomes were as follows: 1) alpha and beta diversity; 2) the changes in intestinal microbial composition. Alpha diversity mainly reflects the diversity of gut microbiota in a sample, which can be expressed as the number of different species in the community (richness) and the evenness in the distribution of individuals in the community (evenness) (Liou et al., 2019). Among them, Shannon index, Pielou index and PD whole tree are indicators reflecting microbial diversity, which comprehensively measure the richness and evenness of bacterial microbiota. The Chao1 index and the number of observed *species* (*sp.*)/operational taxonomic units (OTUs) indicate microbial richness. Beta diversity reflects the differences between before and after VPZ treatments in microbial community composition. Commonly used algorithms are Bray-Curtis, unweighted UniFrac, weighted UniFrac, and Spearman.

### Quality assessment

Two independent investigators (Ouyang ML and Zhao YZ) evaluated the included studies using the risk of bias in non-randomized studies of interventions (ROBINS-I) tool (Sterne et al., 2016). This tool was assessed by seven bias domains: confounding, selection of participants, classification of interventions, deviations from intended interventions, missing data, measurement of outcomes, and selection of the reported result. The overall judgment of risk of bias was classified as low risk, moderate risk, serious risk, critical risk, and no information.

### Subgroup analysis

Considering the different follow-up times of the studies, the pooled analyses of each study were performed at the same follow-up time in order to balance the possible effects of different follow-up times. Therefore, we performed meta-analysis according to three different follow-up times (T < 1 month, 1 month < T < 3 months, and T > 3 months). We then analyzed the results of Shannon index and microbial changes in different subgroups according to country (China vs. Japan), types of therapy (VA-dual vs. VAC-triple vs. VPZ), and participants (Teenagers vs. Adults).

### Statistical analysis

For qualitative data analysis, we counted the number of studies reporting statistically significant differences between before and after treatment for each outcome. The *p*-value < 0.05 was considered statistically significant.

Mean difference (MD) and 95% confidence interval (CI) were used to evaluate the difference of gut microbiota diversity index and relative abundance between VPZ treatments and non-VPZ treatments by the meta-analysis. If a study reported only the sample median and interquartile range (IQR) in continuous data, the sample mean and standard deviation (SD) were approximately estimated using calculation (Shi et al., 2020). In addition, the heterogeneity among the studies was assessed by the *I* ^*2*^ statistic; if *I* ^*2*^ > 50%, the high heterogeneity was considered among the studies. If there was a high heterogeneity, we used a random-effects model for meta-analysis (Higgins et al., 2003). All the statistical process and results visualization were conducted by GraphPad Prism (version 9.0), Stata (version 17.0), RStudio (version 4.3.1) and SPSS (version 24.0).

## Results

### Characteristics of studies

**FIGURE 1:**
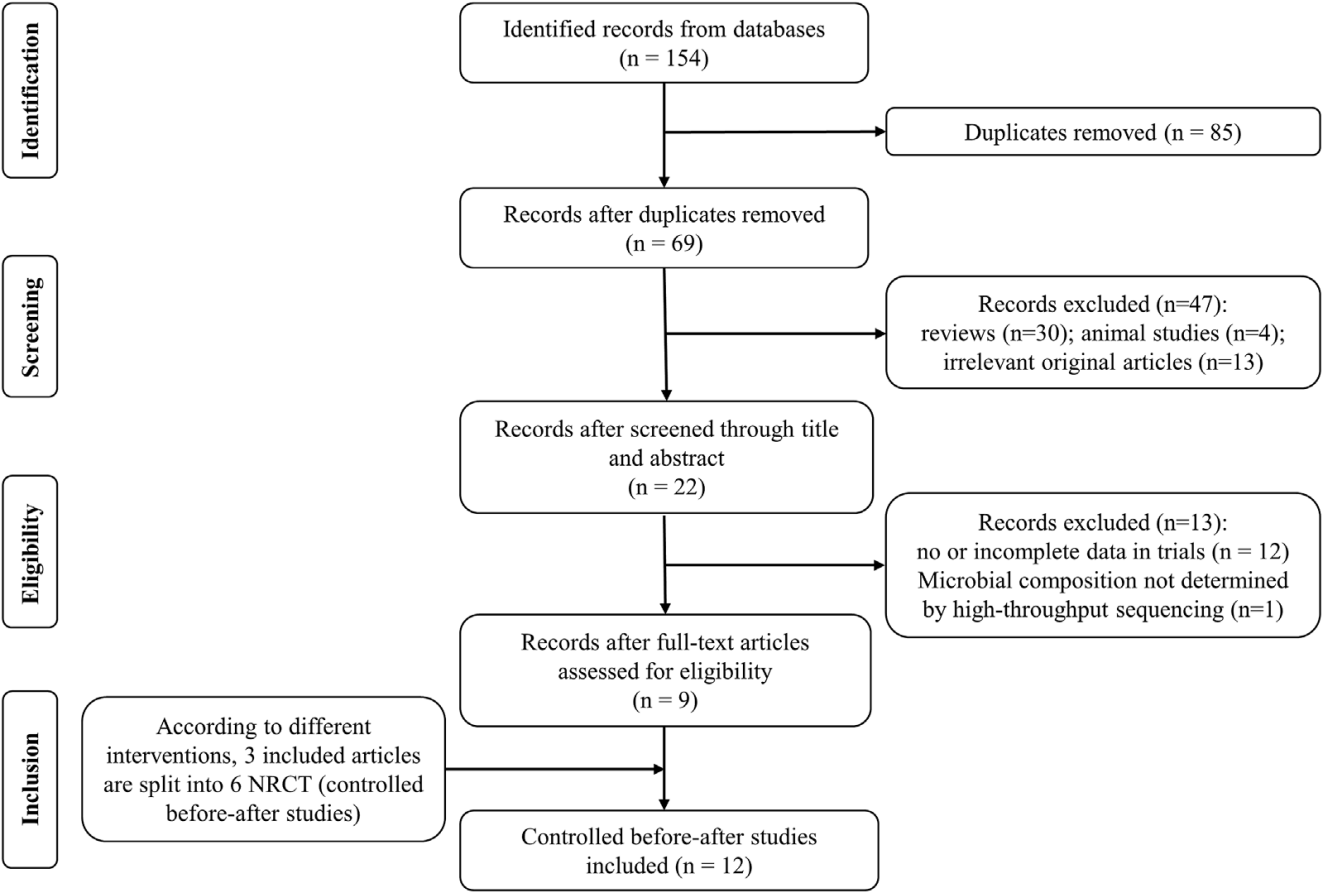
Flow diagram of literature identification and selection process. NRCT: non-randomized controlled trial.

The process of study selection was displayed in Figure 1. From the 154 articles retrieved in the database, we screened nine articles (Otsuka et al., 2017; Gotoda et al., 2018; Kakiuchi et al., 2020; Kakiuchi et al., 2021; Horii et al., 2021; Suzuki et al., 2021; Hu et al., 2022a; Furune et al., 2022; Hu et al., 2023). Three included articles (Horii et al., 2021; Suzuki et al., 2021; Hu et al., 2022a) in last step were split into six substudies according to different treatment measures. Finally, 12 studies were included according with all inclusion criteria in our meta-analysis.

The detailed characteristics of included studies are shown in Table 1. Three studies were conducted in China, and other nine studies were completed in Japan. One studies included participants without *H. pylori* infection, and the others enrolled patients with *H. pylori* infection. Three studies focused on teenagers and the remaining nine studies enrolled adults. Regarding the types of therapy, one studies evaluated VPZ monotherapy, five studies that focused on VA-dual therapy, and six studies that evaluated VAC-triple therapy. All the studies reported the use of 16S ribosomal gene amplicon sequencing from fecal samples for gut microbiota analysis. The amplified region was V3-V4 in eleven studies, one studies did not report the amplified region. All studies used the Illumina sequencing platform. Therefore, this may reduce the risk of detection bias.

**TABLE 1:** The basic characteristics of included studies.

Assessment ID	Country	Participants	Therapy	Follow-up	Sample size	DNA extraction method (region amplified)	Sequencing platform	Database	Outcomes
Furune et al. (2022)	Japan	Adults	VAC-triple	8 weeks/24 weeks	15	the DNeasy PowerSoil Kit (16SrRNA V3-V4)	Illumina Miseq	Greengenes	abcd
Gotoda et al. (2018)	Japan	Teenagers	VAC-triple	1 week/8 weeks	8	NA (16SrRNA V3-V4)	Illumina Miseq	Greengenes	abc
Horii et al. (2021)	Japan	Adults	VAC-triple	1 week/8 weeks	24	brush-type stool collection kit (16SrRNA V3-V4)	Illumina Miseq	Greengenes	abcd
Horii et al. (2021)	Japan	Adults	VA-dual	1 week/8 weeks	19	brush-type stool collection kit (16SrRNA V3-V4)	Illumina Miseq	Greengenes	abcd
Hu et al. (2022a)	China	Adults	VA-dual	1 week/4 weeks	30	the OMEGA Soil DNA Kit (16SrRNA V3-V4)	Illumina Miseq	Greengenes	abcd
Hu et al. (2022a)	China	Adults	VA-dual	1 week/4 weeks	23	the OMEGA Soil DNA Kit (16SrRNA V3-V4)	Illumina Miseq	Greengenes	abcd
Kakiuchi et al. (2020)	Japan	Teenagers	VAC-triple	1 week/8–12 weeks	26	NucleoSpin Microbial DNA Kit (16SrRNA V3-V4)	Illumina Miseq	Greengenes	ad
Kakiuchi et al. (2021)	Japan	Teenagers	VAC-triple	1 week/12 weeks	31	NucleoSpin Microbial DNA Kit (16SrRNA V3-V4)	Illumina Miseq	Greengenes	abcd
Otsuka et al. (2017)	Japan	Adults	VPZ	4 weeks	9	NA	Illumina Miseq	KEGG	abd
Suzuki et al. (2021)	Japan	Adults	VAC-triple	1 year	21	brush-type stool collection kit (16SrRNA V3-V4)	Illumina Miseq	Greengenes	abcd
Suzuki et al. (2021)	Japan	Adults	VA-dual	1 year	18	brush-type stool collection kit (16SrRNA V3-V4)	Illumina Miseq	Greengenes	abcd
Hu et al. (2023)	China	Adults	VA-dual	4 weeks	10	MagPure Stool DNA KF kit B (16SrRNA V3-V4)	Illumina Miseq	KEGG	acd

VPZ: vonoprazan; VAC-triple: vonoprazan, amoxicillin and clarithromycin; VA-dual: vonoprazan and amoxicillin; a: alpha-diversity index; b: beta-diversity; c: microbiome analysis at phylum level; d: microbiome analysis at genus level.

### Quality assessment

Quality assessment was performed among each included study by ROBINS-I. Our results showed that the domain with the lowest risk of bias was the selection of participants and classification of intervention. As for the overall risk of bias, only one article was ranked low, seven articles were of moderate concern, and one was considered serious (Supplementary Figures S1, S2).

### Bacterial diversity

**FIGURE 2:**
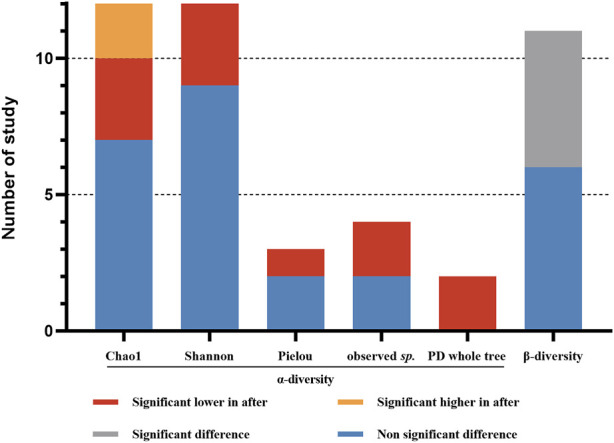
Qualitative analysis for alpha- diversity and beta-diversity before and after VPZ treatment. Observed *sp.*: observed species.

The aim of this study is to investigate the changes of gut microbiota in participants with VPZ use. The result of qualitative analysis of microbial diversity indices before and after VPZ treatments are shown in Figure 2. Three studies reported a significant decrease in the Chao1 index of gut microbiota after VPZ treatments compared to baseline (Kakiuchi et al., 2020; Kakiuchi et al., 2021; Horii et al., 2021), while two studies reported a significant increase after VPZ treatments (Suzuki et al., 2021). For the Pielou index, one study found a significant decrease after VPZ treatments (Hu et al., 2022a), and two studies showed no significant changes (Hu et al., 2022a; Furune et al., 2022). Among the twelve studies that reported the Shannon index, three studies found a significant decrease after VPZ treatments (Kakiuchi et al., 2020; Kakiuchi et al., 2021; Horii et al., 2021). Both two studies reported significant decreased in observed *sp.* and PD whole tree after VPZ treatments (Kakiuchi et al., 2020; Kakiuchi et al., 2021). Significant differences in beta diversity were also observed compared to baseline in five studies (Otsuka et al., 2017; Horii et al., 2021; Suzuki et al., 2021; Hu et al., 2022a). Seven of eleven studies examined beta diversity by computing the weighted and unweighted UniFrac distance between the samples. Two studies analyzed beta diversity using the Bray-Curtis metrics. One study analyzed beta diversity using the unweighted UniFrac metrics. One study analyzed beta diversity using the weighted UniFrac metrics.

**FIGURE 3:**
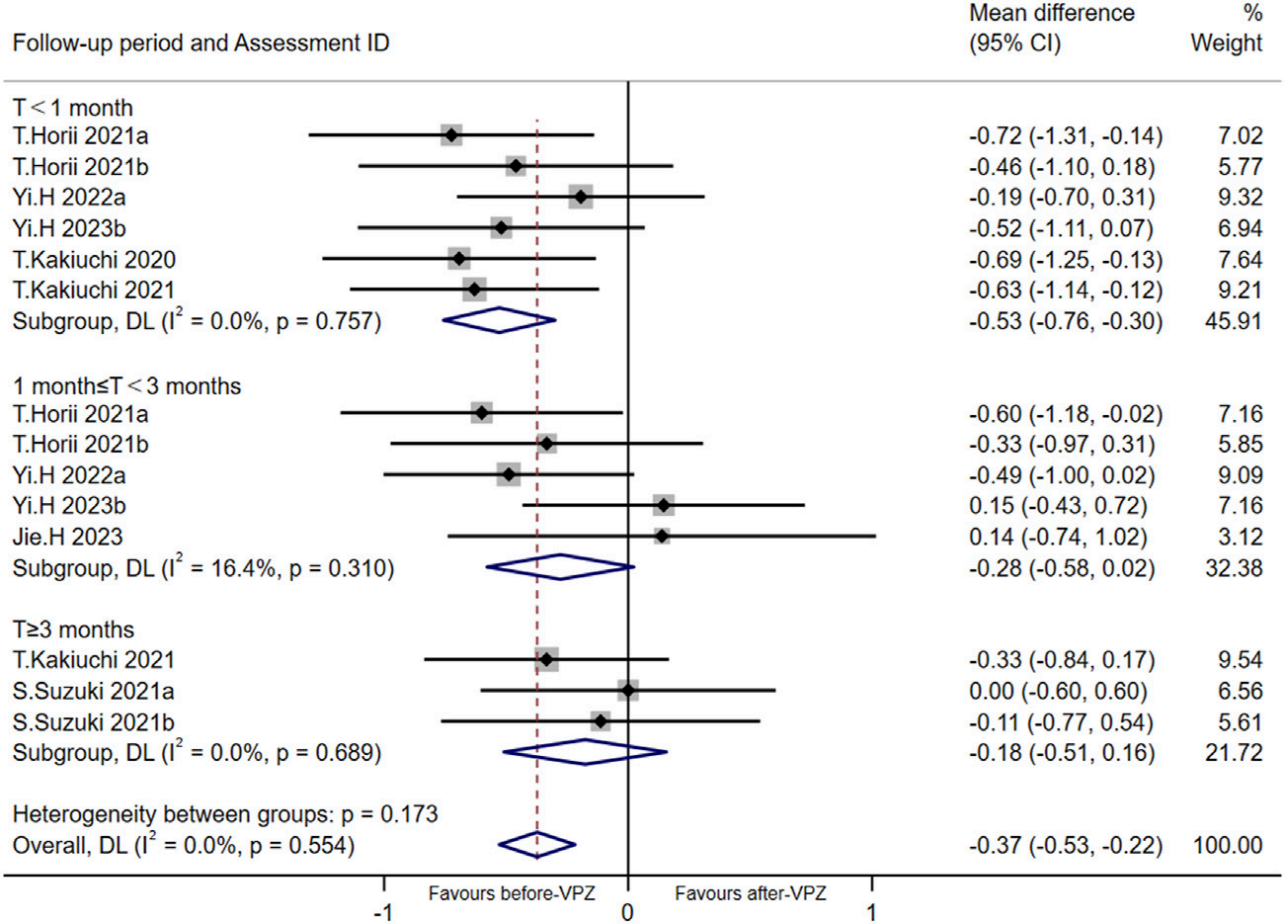
Meta-analysis for Shannon index according to follow-up period. T < 1 month: follow-up time less than 1 month; 1 month ≤ T < 3 months: follow-up time 1-3 months; T ≥ 3 months: follow-up time over 3 months.

Based on the data available for α-diversity index, we performed a meta-analysis of Shannon index according to follow-up periods (Figure 3). The Shannon index decreased significantly in the follow-up period <1 month (MD = −0.53, 95%CI −0.76 to −0.30; *p* < 0.001; *I* ^*2*^ = 0.0%). However, there was no significant differences in Shannon index after VPZ treatments between 1 and 3 months (MD = −0.28, 95%CI −0.58 to 0.02; *p* = 0.070; *I* ^2^ = 16.4%) and more than 3 months (MD = −0.18, 95%CI −0.51 to 0.16; *p* = 0.299; *I* ^2^ = 0.0%) of follow-up periods. We performed an Egger’s test for Shannon diversity (*p* = 0.198) and found the risk of bias was non-significant.

### Microbial changes at the phylum level

**FIGURE 4:**
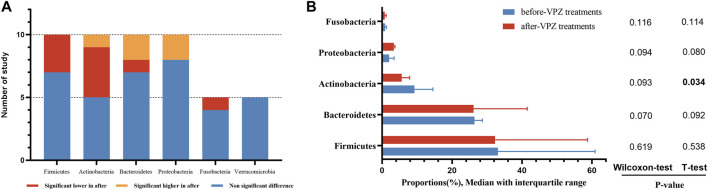
Qualitative and quantitative analysis of gut microbiota at the phylum level. **(A)** Qualitative analysis; **(B)** T-test and Wilcoxon rank-sum test of average abundances at the phylum level.

The qualitative comparison of microbial composition at the phylum level between before and after VPZ treatments is shown in Figure 4A. We analyzed data at the phylum level (including *Firmicutes, Bacteroidetes*, *Actinobacteria*, *Proteobacteria*, and *Fusobacteria*). For *Firmicutes*, in ten studies, seven studies did not observe significant differences between before and after treatment in relative abundance, but three studies observed a significant reduction in its relative abundance after VPZ treatments (Horii et al., 2021; Suzuki et al., 2021; Hu et al., 2022a). In the ten studies, four studies found a reduction in the relative abundance of *Actinobacteria* after VPZ treatment (Gotoda et al., 2018; Horii et al., 2021; Kakiuchi et al., 2021; Hu et al., 2022a), while one study found an increase after VPZ treatment (Hu et al., 2022a). The relative abundances of *Bacteroidetes* showed a significant reduction in one study (Hu et al., 2022a), however, two studies indicated a significant increase (Gotoda et al., 2018; Horii et al., 2021). Compared with the before-VPZ use group, *Proteobacteria* was shown to be enriched in VPZ use group in two studies (Hu et al., 2022a). Regarding the phylum *Fusobacteria*, four studies found no difference in their relative abundance between before and after VPZ treatments, whereas one study found a lower proportion of *Fusobacteria* in VPZ use group (Hu et al., 2022a). The relative abundances of *Verrucomicrobia* did not show differences according to the results of five studies.

We also compared the average abundance of phylum among the included studies by the paired sample t-test and *Wilcoxon* rank-sum test. The abundance of *Actinobacteria* was decreased significantly after VPZ treatment. The abundance of *Proteobacteria* was increased after treatment, but did not reach a significance level (Figure 4B).

**FIGURE 5:**
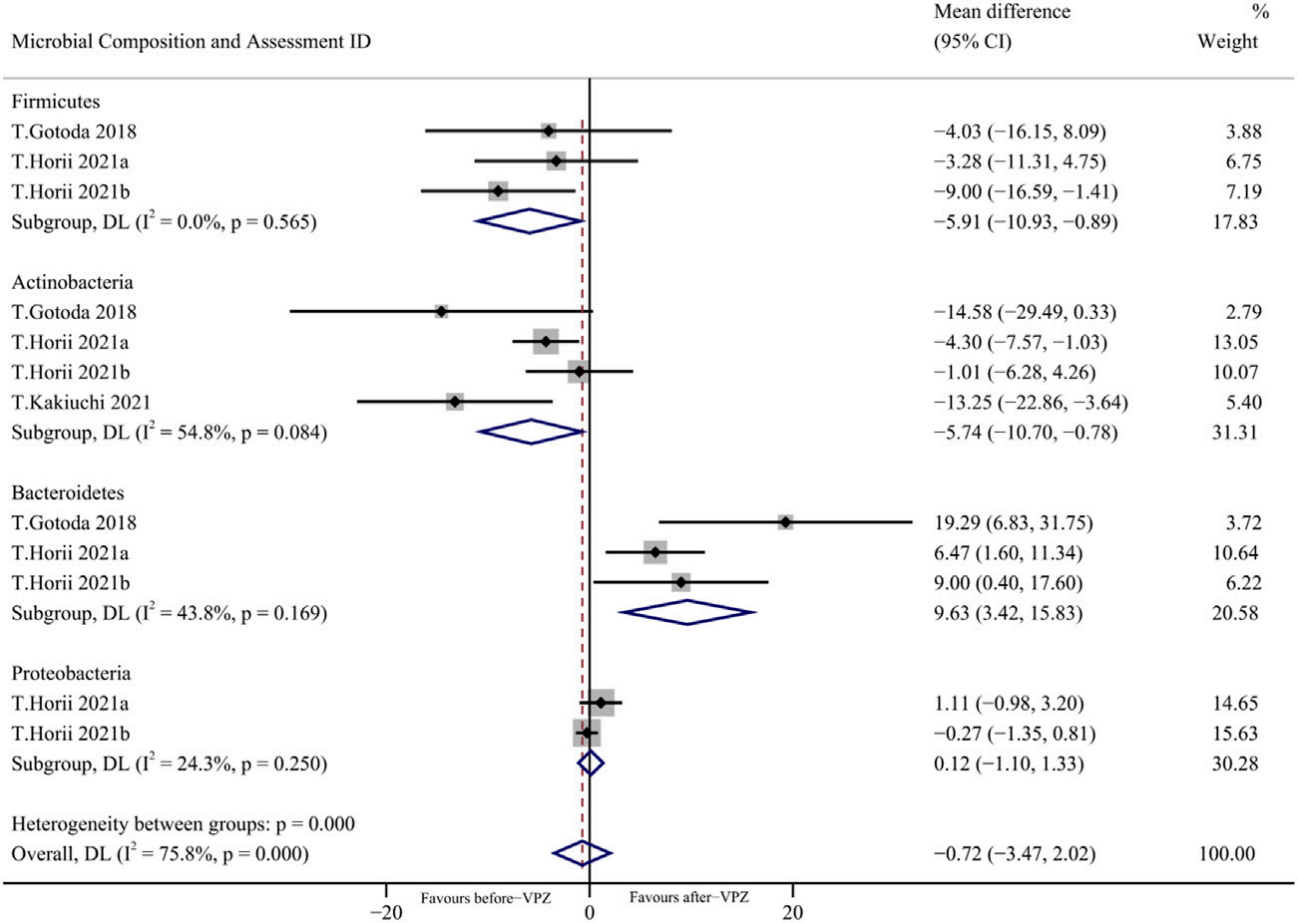
Forest plots of changes in gut microbiota within 1 month after VPZ treatment at the phylum level.

Based on the available evidence, we performed a meta-analysis on the changes of gut microbiota at the phylum level in follow-up periods less than 1 month (Figure 5). We found a significant decrease in the relative abundance of *Firmicutes* (MD = −5.91, 95%CI −10.93 to −0.89; *I* ^*2*^ = 0.0%, n = 3) and *Actinobacteria* (MD = −5.74, 95%CI −10.70 to −0.78, *I* ^2^ = 54.8%; n = 4) and a significant increase in the relative abundance of *Bacteroidetes* (MD = 9.63, 95% CI 3.42 to 15.83; *I* ^2^ = 43.8%, n = 3). Furthermore, we also performed a meta-analysis of the changes in gut microbiota at the phylum level 1–3 months and more than 3 months after VPZ treatment (Supplementary Figures S3, S4).

### Microbial changes at the genus level

**FIGURE 6:**
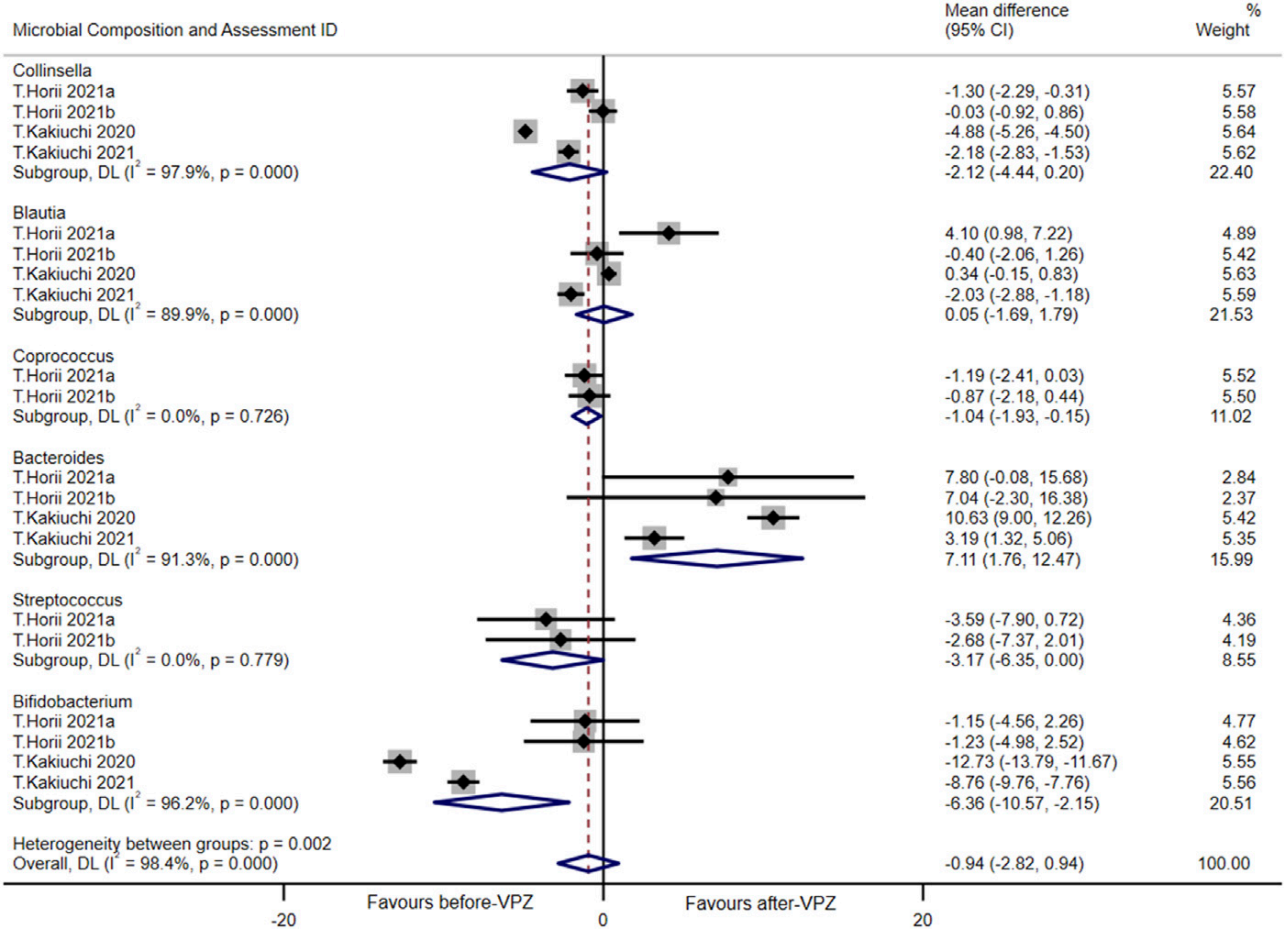
Forest plots of changes in gut microbiota within 1 month after VPZ treatment at the genus level.

A meta-analysis was performed at the genus level (including *Collinsella*, *Blautia*, *Coprococcus*, *Bacteroides*, *Streptococcus*, *Bifidobacterium*) in follow-up periods less than 1 month and statistically significant differences were found between the groups before and after VPZ treatment. Compared with before-VPZ treatment, *Coprococcus* (MD = −1.04, 95%CI −1.93 to −0.15; *I* ^2^ = 0.0%, n = 2) and *Bifidobacterium* (MD = −6.36, 95%CI −10.57 to −2.15; *I* ^2^ = 96.2%, n = 4) were found to have a lower proportion after VPZ treatments. The *Bacteroides* (MD = 7.11, 95%CI 1.76 to 12.47; *I* ^2^ = 91.3%, n = 4) were found to have higher abundances after VPZ treatments than before (Figure 6). In addition, we also performed a meta-analysis of the changes in gut microbiota at the genus level 1–3 months after VPZ treatment (Supplementary Figure S5).

### Subgroup analysis

In the subgroup analysis based on country, we found a significant decrease in the Shannon index in Japan, while there was no significant difference in China. The results of subgroup analyses according to types of therapy and participants were consistent with the overall results. Interestingly, we found that the Shannon index decreased significantly in the short term, and gut microbiota gradually returned to the baseline level with the extension of follow-up time. In the follow-up period ≥3 months, there was no significant difference in the change of bacterial microbiota compared with that before treatment (Supplementary Table S3–S5).

In the subgroup analysis at the phylum level and genus level for different countries, we also found that there was no significant difference in the changes of gut microbiota in China. In the subgroup analysis based on treatment type, compared with VAC-triple therapy, VA-dual therapy showed little difference in the changes of gut microbiota. When VPZ monotherapy appeared, *Blautia* and *Coprococcus* showed significant reduction after treatments. The abundance of *Streptococcus* was significantly increased in VPZ monotherapy, oppositely, significantly decreased in VAC-triple therapy. Within the subgroup of participants, we found reductions in specific bacterial populations in teenagers as compared with adults. The abundance of *Collinsella* and *Bifidobacterium* were significantly decreased in teenagers. From the follow-up period, part of the gut microbiota changed in the short term (T ≤ 1 month and 1 month ≤ T < 3 months) and returned to the baseline level in the long-term (T ≥ 3 months) follow-up. (Supplementary Table S3–S5).

## Discussion

The changes of gut microbiota caused by acid-suppressive drugs potentially increase the risk of adverse reactions, such as small intestinal bacterial overgrowth, inflammatory bowel disease and CDI (Malfertheiner et al., 2017; Oshima et al., 2018; Xia et al., 2021). In this study, we conducted a qualitative analysis and meta-analysis to analyze the effects of vonoprazan on human gut microbial diversity and bacterial composition by comparing the differences before and after vonoprazan treatments. In addition, one thing that’s very important to note: microbiota analysis based on stool sampling is representative of the distal colonic microbiota, but not of the proximal colonic or the ileal microbiota (Falony et al., 2018).

To the best of our knowledge, this is the first study to examine the changes in the gut microbiota after VPZ treatment. Our study presented the following results: 1) Compared to baseline, the Shannon index significantly reduced after VPZ treatments; 2) Based on the results of subgroup analysis, VA-dual therapy had less impact on the gut microbiota than VAC-triple therapy; 3) There was a significant change in the abundance of fecal microbiota at the phylum and genus levels 1 week after VPZ treatments compared to the baseline; 4) The differences in gut microbiota among the groups decreased over a long period of time after treatment with VPZ-containing regimens; 5) There were differences in the changes of gut microbiota after VPZ treatment between different countries and populations.

Our findings are consistent with those of a previous population-based cohort study from the Netherlands in which acid-inhibitory drug use was associated with a decrease in gut microbiota diversity (Imhann et al., 2016). The reduced diversity of gut microbial means that pathogenic species are relatively increased, leading to an unhealthy state (Claesson et al., 2017). Despite the dramatic changes in gut microbiota immediately after VPZ treatment, according to our subgroup analysis, alpha-diversity levels returned to the baseline after 3 months of follow-up.

We observed a reduction in the *Coprococcus* in our meta-analysis, which was consistent with previous studies reporting that PPI use reduces the relative abundance of Lachnospiraceae (Chen et al., 2018). *Coprococcus,* an important member of Lachnospiraceae family of *Firmicutes,* is also an important genus of intestinal bacteria and one of the important producers of butyric acid (Singh et al., 2022). Studies have shown that butyric acid, as a main component of short-chain fatty acids (SCFAs), may inhibit *C. difficile* translocation and reduce *C. difficile*-induced inflammation by increasing intestinal epithelial bacterial tight junctions and reducing intestinal permeability (Fachi et al., 2019). In our study, the decreased abundance of *Coprococcus* may lead to a decrease in the production of butyric acid in SCFAs, resulting in an increased risk of *C. difficile* infection (Martinez et al., 2022).

*Bacteroides* was found to be enriched after VPZ treatments by meta-analysis. The *Bacteroides*, an important member of Bacteroidaceae family of *Bacteroidetes,* are neutral bacteria in the gut microbiota. It has been shown to be involved in carbohydrate metabolism and the synthesis of conjugated linoleic acid, which is known to have anti-diabetic, anti-atherosclerotic, anti-obesity and lipid-lowering effects (Jandhyala et al., 2015). However, when *Bacteroides* enter body parts other than the gastrointestinal region, it can cause abscesses and other infections. With an increase in its abundance, it can also occupy the growth space of other bacteria, leading to an imbalance of intestinal microbiota.

Our study showed that VPZ treatment was associated with *Bifidobacterium* reduction, similar to PPI, due to the inhibition of gastric acid secretion and subsequent disruption of the gastric acid barrier (Imhann et al., 2016). *Bifidobacterium*, an important member of Bifidobacteriaceae family of *Actinobacteria,* binds closely to intestinal mucosal epithelial cells through teichoic-acid. Together with *Lactobacillus*, it forms a biological barrier on the surface of the intestinal mucosa to prevent bacterial colonization and invasion (Xiao et al., 2021). In addition, *Bifidobacterium* can acidify the intestine by producing acid and inhibit the growth of pathogenic bacteria such as *Escherichia coli* and *Staphylococcus aureus*. It also protects and repairs the intestinal biological barrier, playing a role in regulating gut microbiota imbalance (Markowiak-Kopeć and Śliżewska, 2020). The decrease in its abundance increases the intestinal permeability, thereby reducing the absorption of water and sodium and leading to the occurrence of diarrhea, which is a common side effect of VPZ (Gong et al., 2023).

Noteworthily, phylum is a very broad taxonomical term, but that these findings reflect the changes in particular genera, such as the changes in *Firmicutes* being due to changes in *Coprococcus*, changes in *Bacteroidetes* being due to changes in *Bacteroides* and changes in *Actinobacteria* being due to changes in *Bifidobacterium*.

Gut microbiota is closely related to host geographic location, genetics, dietary and lifestyle habits. There are significant differences in gut microbiota among people of different races and regions. Even China and Japan, which are both in Asia, have great differences in gut microbiota. These differences may be caused by eating habits and cultural differences, as well as genetic variation that reflects specific functions of the gut microbiota within a given race or country (Chen et al., 2016). In subgroup analyses by age group, reductions in specific bacterial populations were found in adolescents compared to adults. With aging, the abundance of beneficial bacteria in the gut microbiota significantly decreases, while the abundance of *Enterococcus* and *Clostridium species* increases. At present, there are limited studies on the gut microbiota of adolescents. Compared with adults, adolescents may affect the gut microbiota due to diet, intestinal immaturity, immunity and other factors (Simakachorn et al., 2021). Further validation is needed to explore the effects of VPZ on gut microbiota in adolescents.

Potassium-competitive acid blockers may alter gut microbiota through three different mechanisms. First, P-CAB inhibits gastric acid secretion, reducing the acidity of the stomach, and allowing the survival of microorganisms that would otherwise be killed by the gastric environment. This disrupts the intestinal microbiota and increases the risk of intestinal infection (Perry et al., 2020); Second, the inhibition of gastric acid secretion decreases the activation of pepsinogen and reduces the secretion of pancreatic juice, bile and intestinal juice, leading to weakened gastrointestinal digestion ability. Nutrients that are not easily absorbed, such as proteins, may also affect the distribution of intestinal microbiota after entering the intestine. Third, the low intragastric pH resulting from the protective effect of gastric barrier prevents oral bacteria from entering the gut. When acid-suppressive drugs are used, the weakened protective effect of gastric acid barrier can lead to microbiota imbalance and intestinal infection (Weersma et al., 2020; Hu et al., 2022b).

Given the broad impact of gut microbiota on health and disease, the side effects of acid-suppressive drugs may have important consequences. Therefore, it is worthwhile to attempt to balance VPZ-induced alterations in gut microbiome diversity and composition. In this regard, probiotics have been shown to improve intestinal barrier function by increasing intestinal immunity, ameliorating the effect of acid-suppressive drugs on intestinal microbiota, and also effectively preventing infections and diarrhea in VPZ users (Kakiuchi et al., 2020; Singh et al., 2022). Previous studies have shown that probiotic supplementation during anti-*H. pylori* treatment not only effectively improves the *H. pylori* eradication rate but also reduces the overall incidence of treatment-related adverse events (Lü et al., 2016). In addition, dietary regulation can also supplement beneficial bacteria in the human body and improve the imbalance of gut microbiota.

The strength of this study is that we systematically searched and screened the relevant literature on the effects of VPZ use on the gut microbiota. All included studies treated patients with naive *H. pylori* using first-line therapies (VA-dual and VAC-triple), avoiding the effects of prior use of acid-suppressing drugs and antibiotics. In addition, all stool samples were analyzed by 16S rRNA gene sequencing, and most of the amplified regions were V3-V4, all studies used the Illumina Miseq sequencing platform, which greatly reduced the risk of detection bias and made the results comparable between different studies. Although the results of our study were confounded by antibiotics, clarithromycin increased the abundance of *Firmicutes* based on the effects of antibiotics in other studies (Yang et al., 2021). However, our study showed opposite results, indicating that VPZ might affect the gut microbiota. Based on the existing literature included in this study, the findings are still controversial. The discrepancy between the conflicting results may be due to differences in study populations, interventions, etc. Moreover, there are few studies specifically investigating the effect of VPZ on gut microbiota disorders, and further well-designed studies are needed to address this issue. In addition, the extent to which the new potassium-competitive acid blockers affect the gut microbiota compared with proton pump inhibitors is another concern.

Our study has some limitations. First, the heterogeneity of the included studies was high due to differences in countries, participants, and interventions. Although we performed subgroup analyses, the results were inconsistent. More homogeneous studies are needed to explore the effects of vonoprazan on gut microbiota. Second, because some studies did not report sufficient data to quantitatively analyze the diversity and relative abundance of gut microbiota, the results of the meta-analysis may be biased and cannot fully reflect the potential differences in gut microbiota. Third, because the results of the alpha diversity index for five studies were presented by median and interquartile range, this part of the data needed to be estimated as the mean and standard deviation by formula, which may lead to measurement bias. Since the beta diversity results are presented in the form of graphs, we did not have access to the relevant data, so we could only perform a qualitative analysis based on the results of the included studies. Fourth, the effects of *H. pylori* infection and antibiotics on the gut microbiota were existed in the included studies, resulting in high heterogeneity of the results. More studies are needed to clarify the effects of VPZ on gut microbiota when applied in other acid-related diseases.

## Conclusion

In conclusion, this study analyzed the changes in microbial diversity and bacterial composition before and after vonoprazan treatments. We found a significant reduction in alpha diversity with vonoprazan treatments, which gradually returned to baseline levels with longer follow-up. We also found significant reductions in the abundance of *Coprococcus* and *Bifidobacterium*, which may increase the risk of diseases such as *C. difficile* infection. Due to the large heterogeneity, further studies are needed to confirm these results.

## Data Availability

The original contributions presented in the study are included in the article/Supplementary Material, further inquiries can be directed to the corresponding author.
