# Supplementary material for: Effect of potassium-competitive acid blockers on human gut microbiota: a systematic review and meta-analysis
Source: Front Pharmacol. 2023 Dec 13;14:1269125. doi: 10.3389/fphar.2023.1269125 (PMC10773775; doi:10.3389/fphar.2023.1269125)
Supplement: Supplementary file 1 [file DataSheet1.docx]

***Supplementary Material***

1. **Supplementary Tables**

**Supplementary Table 1. PRISMA checklist**

| **Section and Topic** | **Item #** | **Checklist item** | **Location where item is reported** |
| --- | --- | --- | --- |
| **TITLE** | | |  |
| Title | 1 | Identify the report as a systematic review. | Page 1 |
| **ABSTRACT** | | |  |
| Abstract | 2 | See the PRISMA 2020 for Abstracts checklist. | Page 1 |
| **INTRODUCTION** | | |  |
| Rationale | 3 | Describe the rationale for the review in the context of existing knowledge. | Page 2 |
| Objectives | 4 | Provide an explicit statement of the objective(s) or question(s) the review addresses. | Page 2 |
| **METHODS** | | |  |
| Eligibility criteria | 5 | Specify the inclusion and exclusion criteria for the review and how studies were grouped for the syntheses. | Page 2 |
| Information sources | 6 | Specify all databases, registers, websites, organisations, reference lists and other sources searched or consulted to identify studies. Specify the date when each source was last searched or consulted. | Page 2 |
| Search strategy | 7 | Present the full search strategies for all databases, registers and websites, including any filters and limits used. | Supplementary Table 2 |
| Selection process | 8 | Specify the methods used to decide whether a study met the inclusion criteria of the review, including how many reviewers screened each record and each report retrieved, whether they worked independently, and if applicable, details of automation tools used in the process. | Page 2 |
| Data collection process | 9 | Specify the methods used to collect data from reports, including how many reviewers collected data from each report, whether they worked independently, any processes for obtaining or confirming data from study investigators, and if applicable, details of automation tools used in the process. | Page 2 |
| Data items | 10a | List and define all outcomes for which data were sought. Specify whether all results that were compatible with each outcome domain in each study were sought (e.g. for all measures, time points, analyses), and if not, the methods used to decide which results to collect. | Page 2 |
|  | 10b | List and define all other variables for which data were sought (e.g. participant and intervention characteristics, funding sources). Describe any assumptions made about any missing or unclear information. | Page 2-3 |
| Study risk of bias assessment | 11 | Specify the methods used to assess risk of bias in the included studies, including details of the tool(s) used, how many reviewers assessed each study and whether they worked independently, and if applicable, details of automation tools used in the process. | Page 3 |
| Effect measures | 12 | Specify for each outcome the effect measure(s) (e.g. risk ratio, mean difference) used in the synthesis or presentation of results. | Page 3 |
| Synthesis methods | 13a | Describe the processes used to decide which studies were eligible for each synthesis (e.g. tabulating the study intervention characteristics and comparing against the planned groups for each synthesis (item #5)). | Page 3 |
|  | 13b | Describe any methods required to prepare the data for presentation or synthesis, such as handling of missing summary statistics, or data conversions. | Page 3 |
|  | 13c | Describe any methods used to tabulate or visually display results of individual studies and syntheses. | Page 3 |
|  | 13d | Describe any methods used to synthesize results and provide a rationale for the choice(s). If meta-analysis was performed, describe the model(s), method(s) to identify the presence and extent of statistical heterogeneity, and software package(s) used. | Page 3 |
|  | 13e | Describe any methods used to explore possible causes of heterogeneity among study results (e.g. subgroup analysis, meta-regression). | Page 3 |
|  | 13f | Describe any sensitivity analyses conducted to assess robustness of the synthesized results. | / |
| Reporting bias assessment | 14 | Describe any methods used to assess risk of bias due to missing results in a synthesis (arising from reporting biases). | Page 3 |
| Certainty assessment | 15 | Describe any methods used to assess certainty (or confidence) in the body of evidence for an outcome. | / |
| **RESULTS** | | |  |
| Study selection | 16a | Describe the results of the search and selection process, from the number of records identified in the search to the number of studies included in the review, ideally using a flow diagram. | Page 3-4, Figure 1 |
|  | 16b | Cite studies that might appear to meet the inclusion criteria, but which were excluded, and explain why they were excluded. | Figure 1 |
| Study characteristics | 17 | Cite each included study and present its characteristics. | Page 3, Table 1 |
| Risk of bias in studies | 18 | Present assessments of risk of bias for each included study. | Page 3, Supplementary Figure 1, 2 |
| Results of individual studies | 19 | For all outcomes, present, for each study: (a) summary statistics for each group (where appropriate) and (b) an effect estimate and its precision (e.g. confidence/credible interval), ideally using structured tables or plots. | Page 3-5  Table 1 |
| Results of syntheses | 20a | For each synthesis, briefly summarise the characteristics and risk of bias among contributing studies. | Page 4-6 |
|  | 20b | Present results of all statistical syntheses conducted. If meta-analysis was done, present for each the summary estimate and its precision (e.g. confidence/credible interval) and measures of statistical heterogeneity. If comparing groups, describe the direction of the effect. | Page 4-6 |
|  | 20c | Present results of all investigations of possible causes of heterogeneity among study results. | Page 4-6 |
|  | 20d | Present results of all sensitivity analyses conducted to assess the robustness of the synthesized results. | Supplementary Table 3-5 |
| Reporting biases | 21 | Present assessments of risk of bias due to missing results (arising from reporting biases) for each synthesis assessed. | Page 4-6 |
| Certainty of evidence | 22 | Present assessments of certainty (or confidence) in the body of evidence for each outcome assessed. | Page 4-6 |
| **DISCUSSION** | | |  |
| Discussion | 23a | Provide a general interpretation of the results in the context of other evidence. | Page 6-9 |
|  | 23b | Discuss any limitations of the evidence included in the review. | Page 9 |
|  | 23c | Discuss any limitations of the review processes used. | Page 9 |
|  | 23d | Discuss implications of the results for practice, policy, and future research. | Page 6-9 |
| **OTHER INFORMATION** | | |  |
| Registration and protocol | 24a | Provide registration information for the review, including register name and registration number, or state that the review was not registered. | Page 1-2 |
|  | 24b | Indicate where the review protocol can be accessed, or state that a protocol was not prepared. | Page 1-2 |
|  | 24c | Describe and explain any amendments to information provided at registration or in the protocol. | / |
| Support | 25 | Describe sources of financial or non-financial support for the review, and the role of the funders or sponsors in the review. | Page 9 |
| Competing interests | 26 | Declare any competing interests of review authors. | Page 9 |
| Availability of data, code and other materials | 27 | Report which of the following are publicly available and where they can be found: template data collection forms; data extracted from included studies; data used for all analyses; analytic code; any other materials used in the review. | Page 9 |

*From:*  Page MJ, McKenzie JE, Bossuyt PM, Boutron I, Hoffmann TC, Mulrow CD, et al. The PRISMA 2020 statement: an updated guideline for reporting systematic reviews. BMJ 2021;372:n71. doi: 10.1136/bmj.n71

**Supplementary Table 2. Search strategy**

1. Search Strategy for PubMed

| ID | Search |
| --- | --- |
| #1 | "vonoprazan"[Title/Abstract] OR "tak-438"[Title/Abstract] OR "tak438"[Title/Abstract] OR "tak-438"[Title/Abstract] OR "tegoprazan"[Title/Abstract] OR "CJ-12420"[Title/Abstract] OR "revaprazan"[Title/Abstract] OR "keverprazan"[Title/Abstract] OR "KFP-H008"[Title/Abstract] OR "potassium competitive acid blocker"[Title/Abstract] OR "potassium competitive acid blockers"[Title/Abstract] OR "P-CAB"[Title/Abstract] |
| #2 | ((((((((((((((((((((((((((((((((((((("Gastrointestinal Microbiome"[Mesh]) OR (Gastrointestinal Microbiomes[Title/Abstract])) OR (Microbiome, Gastrointestinal[Title/Abstract])) OR (Gut Microbiome[Title/Abstract])) OR (Gut Microbiomes[Title/Abstract])) OR (Microbiome, Gut[Title/Abstract])) OR (Gut Microflora[Title/Abstract])) OR (Microflora, Gut[Title/Abstract])) OR (Gut Microbiota[Title/Abstract])) OR (Gut Microbiotas[Title/Abstract])) OR (Microbiota, Gut[Title/Abstract])) OR (Gastrointestinal Flora[Title/Abstract])) OR (Flora, Gastrointestinal[Title/Abstract])) OR (Gut Flora[Title/Abstract])) OR (Flora, Gut[Title/Abstract])) OR (Gastrointestinal Microbiota[Title/Abstract])) OR (Gastrointestinal Microbiotas[Title/Abstract])) OR (Microbiota, Gastrointestinal[Title/Abstract])) OR (Gastrointestinal Microbial Community[Title/Abstract])) OR (Gastrointestinal Microbial Communities[Title/Abstract])) OR (Microbial Community, Gastrointestinal[Title/Abstract])) OR (Gastrointestinal Microflora[Title/Abstract])) OR (Microflora, Gastrointestinal[Title/Abstract])) OR (Gastric Microbiome[Title/Abstract])) OR (Gastric Microbiomes[Title/Abstract])) OR (Microbiome, Gastric[Title/Abstract])) OR (Intestinal Microbiome[Title/Abstract])) OR (Intestinal Microbiomes[Title/Abstract])) OR (Microbiome, Intestinal[Title/Abstract])) OR (Intestinal Microbiota[Title/Abstract])) OR (Intestinal Microbiotas[Title/Abstract])) OR (Microbiota, Intestinal[Title/Abstract])) OR (Intestinal Microflora[Title/Abstract])) OR (Microflora, Intestinal[Title/Abstract])) OR (Intestinal Flora[Title/Abstract])) OR (Flora, Intestinal[Title/Abstract])) OR (Enteric Bacteria[Title/Abstract])) OR (Bacteria, Enteric[Title/Abstract]) |
| #3 | #1 AND #2 |

(2) Search Strategy for EMBASE

| #1 | ('potassium competitive acid blocker*' or 'p-cab' or 'vonoprazan' or 'tak?438' or 'tegoprazan' or 'CJ-12420' or 'revaprazan' or 'keverprazan' or 'KFP-H008').mp. [mp=title, abstract, heading word, drug trade name, original title, device manufacturer, drug manufacturer, device trade name, keyword heading word, floating subheading word, candidate term word] |
| --- | --- |
| #2 | ('Gastrointestinal Microbiome' or 'Gastrointestinal Microbiomes' or 'Microbiome, Gastrointestinal' or 'Gut Microbiome' or 'Gut Microbiomes' or 'Microbiome, Gut' or 'Gut Microflora' or 'Microflora, Gut' or 'Gut Microbiota' or 'Gut Microbiotas' or 'Microbiota, Gut' or 'Gastrointestinal Flora' or 'Flora, Gastrointestinal' or 'Gut Flora' or 'Flora, Gut' or 'Gastrointestinal Microbiota' or 'Gastrointestinal Microbiotas' or 'Microbiota, Gastrointestinal' or 'Gastrointestinal Microbial Community' or 'Gastrointestinal Microbial Communities' or 'Microbial Community, Gastrointestinal' or 'Gastrointestinal Microflora' or 'Microflora, Gastrointestinal' or 'Gastric Microbiome' or 'Gastric Microbiomes' or 'Microbiome, Gastric' or 'Intestinal Microbiome' or 'Intestinal Microbiomes' or 'Microbiome, Intestinal' or 'Intestinal Microbiota' or 'Intestinal Microbiotas' or 'Microbiota, Intestinal' or 'Intestinal Microflora' or 'Microflora, Intestinal' or 'Intestinal Flora' or 'Flora, Intestinal' or 'Enteric Bacteria' or 'Bacteria, Enteric').mp. [mp=title, abstract, heading word, drug trade name, original title, device manufacturer, drug manufacturer, device trade name, keyword heading word, floating subheading word, candidate term word] |
| #3 | #1 AND #2 |

(3) Search Strategy for Web of Science

| #1 | TS= (potassium competitive acid blocker* OR p-cab*OR vonoprazan OR tak?438 OR tegoprazan OR CJ-12420 OR revaprazan OR keverprazan OR KFP-H008) |
| --- | --- |
| #2 | TS=(Gastrointestinal Microbiome OR Gastrointestinal Microbiomes OR Microbiome, Gastrointestinal OR Gut Microbiome OR Gut Microbiomes OR Microbiome, Gut OR Gut Microflora OR Microflora, Gut OR Gut Microbiota OR Gut Microbiotas OR Microbiota, Gut OR Gastrointestinal Flora OR Flora, Gastrointestinal OR Gut Flora OR Flora, Gut OR Gastrointestinal Microbiota OR Gastrointestinal Microbiotas OR Microbiota, Gastrointestinal OR Gastrointestinal Microbial Community OR Gastrointestinal Microbial Communities OR Microbial Community, Gastrointestinal OR Gastrointestinal Microflora OR Microflora, Gastrointestinal OR Gastric Microbiome OR Gastric Microbiomes OR Microbiome, Gastric OR Intestinal Microbiome OR Intestinal Microbiomes OR Microbiome, Intestinal OR Intestinal Microbiota OR Intestinal Microbiotas OR Microbiota, Intestinal OR Intestinal Microflora OR Microflora, Intestinal OR Intestinal Flora OR Flora, Intestinal OR Enteric Bacteria OR Bacteria, Enteric) |
| #3 | #1 AND #2 |

(4) Search Strategy for Scopus

| #1 | TITLE-ABS-KEY ("potassium competitive acid blocker*" OR "p-cab*") |
| --- | --- |
| #2 | TITLE-ABS-KEY ("vonoprazan" OR "tak-438") |
| #3 | TITLE-ABS-KEY ("tegoprazan" OR "CJ-12420") |
| #4 | TITLE-ABS-KEY ("revaprazan") |
| #5 | TITLE-ABS-KEY ("keverprazan" OR "KFP-H008") |
| #6 | #1 OR #2 OR #3 OR #4 OR #5 |
| #7 | TITLE-ABS-KEY ("Gastrointestinal Microbiome" OR "Gastrointestinal Microbiomes" OR "Microbiome, Gastrointestinal" OR "Gut Microbiome" OR "Gut Microbiomes" OR "Microbiome, Gut" OR "Gut Microflora" OR "Microflora, Gut" OR "Gut Microbiota" OR "Gut Microbiotas" OR "Microbiota, Gut" OR "Gastrointestinal Flora" OR "Flora, Gastrointestinal" OR "Gut Flora" OR "Flora, Gut" OR "Gastrointestinal Microbiota" OR "Gastrointestinal Microbiotas" OR "Microbiota, Gastrointestinal" OR "Gastrointestinal Microbial Community" OR "Gastrointestinal Microbial Communities" OR "Microbial Community, Gastrointestinal" OR "Gastrointestinal Microflora" OR "Microflora, Gastrointestinal" OR "Gastric Microbiome" OR "Gastric Microbiomes" OR "Microbiome, Gastric" OR "Intestinal Microbiome" OR "Intestinal Microbiomes" OR "Microbiome, Intestinal" OR "Intestinal Microbiota" OR "Intestinal Microbiotas" OR "Microbiota, Intestinal" OR "Intestinal Microflora" OR "Microflora, Intestinal" OR "Intestinal Flora" OR "Flora, Intestinal" OR "Enteric Bacteria" OR "Bacteria, Enteric") |
| #8 | #6 AND #7 |

(5) Search Strategy for Cochrane Library

| #1 | MeSH descriptor: [potassium competitive acid blocker] explode all trees |
| --- | --- |
| #2 | (p-cab*OR vonoprazan OR tak?438 OR tegoprazan OR CJ-12420 OR revaprazan OR keverprazan OR KFP-H008) Enteric in Title Abstract Keyword - (Word variations have been searched) |
| #3 | #1 OR #2 |
| #4 | MeSH descriptor: [Gastrointestinal Microbiome] explode all trees |
| #5 | (Microbiome, Gastrointestinal OR Gut Microbiome OR Gut Microbiomes OR Microbiome, Gut OR Gut Microflora OR Microflora, Gut OR Gut Microbiota OR Gut Microbiotas OR Microbiota, Gut OR Gastrointestinal Flora OR Flora, Gastrointestinal OR Gut Flora OR Flora, Gut OR Gastrointestinal Microbiota OR Gastrointestinal Microbiotas OR Microbiota, Gastrointestinal OR Gastrointestinal Microbial Community OR Gastrointestinal Microbial Communities OR Microbial Community, Gastrointestinal OR Gastrointestinal Microflora OR Microflora, Gastrointestinal OR Gastric Microbiome OR Gastric Microbiomes OR Microbiome, Gastric OR Intestinal Microbiome OR Intestinal Microbiomes OR Microbiome, Intestinal OR Intestinal Microbiota OR Intestinal Microbiotas OR Microbiota, Intestinal OR Intestinal Microflora OR Microflora, Intestinal OR Intestinal Flora OR Flora, Intestinal OR Enteric Bacteria OR Bacteria, Enteric) Enteric in Title Abstract Keyword - (Word variations have been searched) |
| #6 | #4 OR #5 |
| #7 | #3 AND #6 |

**Supplementary Table 3.** Overall and subgroup data with follow-up time less than 1 month, including Shannon index, the phylum and genus level changes in gut microbiota across different countries, types of therapy and participants in this meta-analysis.

| Variable | Overall data | Country | | Types of therapy | | | Participants | |
| --- | --- | --- | --- | --- | --- | --- | --- | --- |
|  |  | China | Japan | VAC-triple | VA-dual | VPZ | Teenagers | Adults |
| Shannon index | **-0.53 (-0.76, -0.30)** | **-0.33 (-0.72, -0.05)** | **-0.64 (-0.92, -0.35)** | **-0.68 (-0.99, -0.36)** | **-0.37 (-0.70, -0.04)** | — | **-0.66 (-1.04, -0.28)** | **-0.45 (-0.74, -0.17)** |
| Phylum MD (95% CI) |  |  |  |  |  |  |  |  |
| Firmicutes | **-5.91 (-10.93, -0.89)** | — | **-5.91 (-10.93, -0.89)** | -3.51 (-10.20, 3.19) | **-9.00 (-16.59, -1.41)** | — | -4.03 (-16.15, 8.09) | **-6.30 (-11.89, -0.70)** |
| Actinobacteria | **-5.74 (-10.70, -0.78)** | — | **-5.74 (-10.70, -0.78)** | **-8.70 (-16.01, -1.38)** | -1.01 (-6.28, 4.26) | — | **-13.64 (-21.72, -5.56)** | **-3.33 (-6.27, -0.39)** |
| Bacteroidetes | **9.63 (3.42, 15.83)** | — | **9.63 (3.42, 15.83)** | 11.54 (-0.74, 23.83) | **9.00 (0.40, 17.60)** | — | **19.29 (6.83, 31.75)** | **7.08 (2.85, 11.32)** |
| Proteobacteria | -0.12 (-1.10, 1.33) | — | -0.12 (-1.10, 1.33) | 1.11 (-0.98, 3.2) | -0.27 (-1.35, 0.81) | — | — | -0.12 (-1.10, 1.33) |
| Genus MD (95% CI) |  |  |  |  |  |  |  |  |
| Collinsella | -2.12 (-4.44, 0.20) | — | -2.12 (-4.44, 0.20) | **-2.82 (-5.11, -0.52)** | -0.03 (-0.92, 0.86) | — | **-3.54 (-6.19, -0.90)** | -0.65 (-1.89, 0.60) |
| Blautia | 0.05 (-1.69, 1.79) | — | 0.05 (-1.69, 1.79) | 0.29 (-1.95, 2.52) | -0.40 (-2.06, 1.26) | — | -0.82 (-3.14, 1.50) | 1.65 (-2.74, 6.04) |
| Lachnospira | -0.42 (-0.95, 0.12) | -0.40 (-2.15, 1.35) | -0.45 (-1.25, 0.35) | -0.08 (-0.79, 0.63) | **-0.80 (-1.57, -0.03)** | — | — | -0.42 (-0.95, 0.12) |
| Coprococcus | **-1.04 (-1.93, -0.15)** | — | **-1.04 (-1.93, -0.15)** | -1.19 (-2.41, 0.03) | -0.87 (-2.18, 0.44) | — | — | **-1.04 (-1.93, -0.15)** |
| Bacteroides | **7.11 (1.76, 12.47)** | — | **7.11 (1.76, 12.47)** | **7.13 (1.07, 13.20)** | 7.04 (-2.30, 16.38) | — | 6.92 (-0.37, 14.22) | **7.48 (1.46, 13.51)** |
| Streptococcus | -3.17 (-6.35, 0.00) | — | -3.17 (-6.35, 0.00) | -3.59 (-7.90, 0.72) | -2.68 (-7.37, 2.01) | — | — | -3.17 (-6.35, 0.00) |
| Bifidobacterium | **-6.36 (-10.57, -2.15)** | — | **-6.36 (-10.57, -2.15)** | **-7.92 (-12.23, -3.61)** | -1.23 (-4.98, 2.52) | — | **-10.74 (-14.63, -6.85)** | -1.19 (-3.71, 1.34) |

MD: Mean difference; CI: Confidence interval; VAC-triple: Vonoprazan, amoxicillin and clarithromycin; VA-dual: Vonoprazan and amoxicillin; VPZ: Vonoprazan.

**Supplementary Table 4.** Overall and subgroup data with follow-up time 1-3 months, including Shannon index, the phylum and genus level changes in gut microbiota across different countries, types of therapy and participants in this meta-analysis.

| Variable | Overall data | Country | | Types of therapy | | | | Participants | | |
| --- | --- | --- | --- | --- | --- | --- | --- | --- | --- | --- |
|  |  | China | Japan | | VAC-triple | VA-dual | VPZ | | Teenagers | Adults |
| Shannon index | -0.28 (-0.58, 0.02) | -0.12 (-0.58, 0.33) | **-0.48 (-0.91, -0.05)** | | **-0.60 (-1.18, -0.02)** | -0.19 (-0.52, 0.14) | — | | — | -0.28 (-0.58, 0.02) |
| Phylum MD (95% CI) |  |  |  | |  |  |  | |  |  |
| Firmicutes | -0.67 (-4.69, 3.34) | 1.00 (-5.84, 7.84) | -1.35 (-5.84, 7.84) | | 0.79 (-6.07, 7.65) | -1.95 (-8.72, 4.83) | — | | 5.59 (-4.95, 16.13) | -1.69 (-5.94, 2.55) |
| Actinobacteria | **-2.70 (-5.14, -0.25)** | — | **-2.70 (-5.14, -0.25)** | | **-3.23 (-6.05, -0.40)** | -1.10 (-6.00, 3.80) | — | | -10.17 (-26.11, 5.77) | **-2.51 (-4.99, -0.04)** |
| Bacteroidetes | 3.08 (-0.43, 6.59) | -1.20 (-13.36, 10.96) | 3.47 (-0.20, 7.13) | | 3.62 (-0.59, 7.82) | 1.85 (-4.52, 8.22) | — | | 6.49 (-4.20, 17.18) | 2.67 (-1.05, 6.38) |
| Proteobacteria | -0.45 (-1.14, 0.23) | 0.10 (-1.15, 1.35) | -0.69 (-1.51, 0.13) | | -0.79 (-2.00, 0.42) | -0.29 (-1.13, 0.54) | — | | — | -0.45 (-1.14, 0.23) |
| Genus MD (95% CI) |  |  |  | |  |  |  | |  |  |
| Collinsella | -1.14 (-3.01, 0.74) | — | -1.14 (-3.01, 0.74) | | -1.73 (-3.72, 0.26) | 0.81 (-0.34, 1.96) | -6.34 (-15.39, 2.71) | | **-2.73 (-3.20, -2.26)** | -0.19 (-1.73, 1.35) |
| Blautia | 0.53 (-0.25, 1.31) | 0.10 (-0.17, 0.37) | 0.57 (-1.45, 2.59) | | 1.91 (-1.04, 4.86) | 0.12 (-0.15, 0.39) | **-9.00 (-15.05, -2.95)** | | **0.71 (0.41, 1.01)** | 0.31 (-1.80, 2.41) |
| Lachnospira | -0.42 (-0.95, 0.12) | -0.40 (-2.15, 1.35) | -0.45 (-1.25, 0.35) | | -0.08 (-0.79, 0.63) | **-0.80 (-1.57, -0.03)** | — | | — | -0.42 (-0.95, 0.12) |
| Coprococcus | -0.70 (-1.83, 0.43) | — | -0.70 (-1.83, 0.43) | | -0.02 (-1.49, 1.45) | -0.32 (-1.82, 1.18) | **-1.96 (-3.65, -0.27)** | | — | -0.70 (-1.83, 0.43) |
| Bacteroides | **2.64 (1.67, 3.60)** | 2.30 (-1.44, 6.04) | **2.66 (1.66, 3.66)** | | **2.52 (1.50, 3.54)** | 2.59 (-0.81, 5.98) | **7.69 (1.00, 14.38)** | | **2.49 (1.46, 3.52)** | **3.74 (0.90, 6.59)** |
| Streptococcus | 0.74 (-7.20, 8.67) | — | 0.74 (-7.20, 8.67) | | **-4.78 (-9.03, -0.53)** | -2.82 (-7.33, 1.69) | **13.32 (4.09, 22.55)** | | — | 0.74 (-7.20, 8.67) |
| Bifidobacterium | -3.67 (-8.34, 0.99) | — | -3.67 (-8.34, 0.99) | | -4.68 (-10.66, 1.29) | -1.47 (-5.20, 2.26) | -0.30 (-42.67, 42.07) | | **-7.51 (-8.51, -6.51)** | -1.43 (-3.95, 1.09) |

MD: Mean difference; CI: Confidence interval; VAC-triple: Vonoprazan, amoxicillin and clarithromycin; VA-dual: Vonoprazan and amoxicillin; VPZ: Vonoprazan.

**Supplementary Table 5.** Overall and subgroup data with follow-up time over 3 months, including Shannon index, the phylum and genus level changes in gut microbiota across different countries, types of therapy and participants in this meta-analysis.

| Variable | Overall data | Country | | Types of therapy | | | Participants | |
| --- | --- | --- | --- | --- | --- | --- | --- | --- |
|  |  | China | Japan | VAC-triple | VA-dual | VPZ | Teenagers | Adults |
| Shannon index | -0.18 (-0.51, 0.16) | — | -0.18 (-0.51, 0.16) | -0.20 (-0.58, 0.19) | -0.11 (-0.77, 0.54) | — | -0.33 (-0.84, 0.17) | -0.05 (-0.50, 0.39) |
| Phylum MD (95% CI) |  |  |  |  |  |  |  |  |
| Firmicutes | **-6.35 (-11.09, -1.60)** | — | **-6.35 (-11.09, -1.60)** | -4.60 (-10.48, 1.28) | **-9.60 (-17.63, -1.57)** | — | — | **-6.35 (-11.09, -1.60)** |
| Actinobacteria | -7.09 (-18.24, 4.06) | — | -7.09 (-18.24, 4.06) | -7.09 (-18.24, 4.06) | — | — | -7.09 (-18.24, 4.06) | — |
| Bacteroidetes | — | — | — | — | — | — | — | — |
| Proteobacteria | — | — | — | — | — | — | — | — |
| Genus MD (95% CI) |  |  |  |  |  |  |  |  |
| Collinsella | 0.35 (-0.20, 0.90) | — | 0.35 (-0.20, 0.90) | 0.35 (-0.20, 0.90) | — | — | 0.35 (-0.20, 0.90) | — |
| Blautia | **-1.95 (-2.70, -1.20)** | — | **-1.95 (-2.70, -1.20)** | **-1.95 (-2.70, -1.20)** | — | — | **-1.95 (-2.70, -1.20)** | — |
| Lachnospira | — | — | — | — | — | — | — | — |
| Coprococcus | — | — | — | — | — | — | — | — |
| Bacteroides | **3.42 (2.09, 4.75)** | — | **3.42 (2.09, 4.75)** | **3.42 (2.09, 4.75)** | — | — | **3.42 (2.09, 4.75)** | — |
| Streptococcus | — | — | — | — | — | — | — | — |
| Bifidobacterium | **-4.56 (-5.61, -3.51)** | — | **-4.56 (-5.61, -3.51)** | **-4.56 (-5.61, -3.51)** | — | — | **-4.56 (-5.61, -3.51)** | — |

MD: Mean difference; CI: Confidence interval; VAC-triple: Vonoprazan, amoxicillin and clarithromycin; VA-dual: Vonoprazan and amoxicillin; VPZ: Vonoprazan.

1. **Supplementary Figures**

**Supplementary Figure 1.**

**
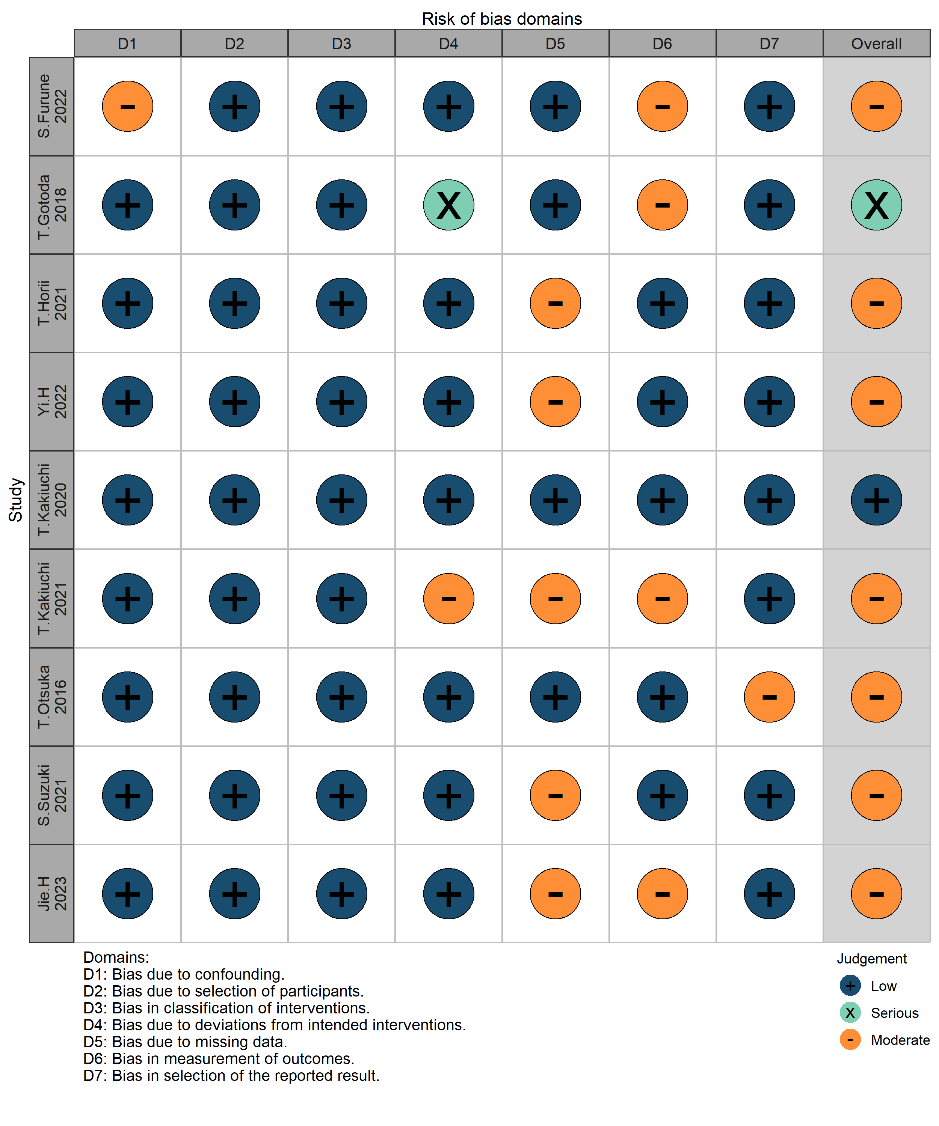
**

**Supplementary Figure 1.** Risk of bias for included studies assessed by ROBINS-I tool.

**Supplementary Figure 2.** **
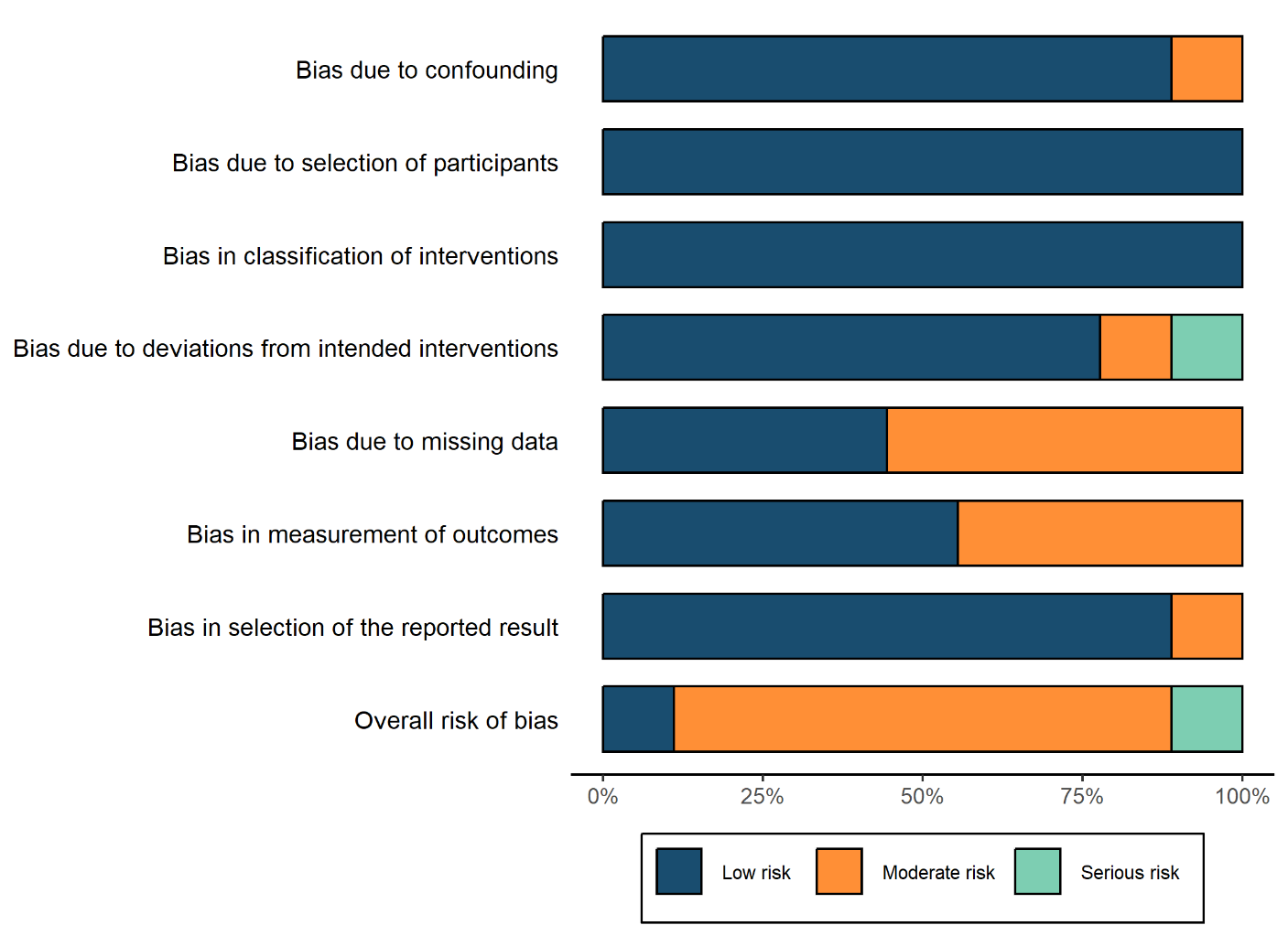
**

**Supplementary Figure 2.** The risk of bias map depicting the proportion of each bias risk item in all studies.

**Supplementary Figure 3.**

**
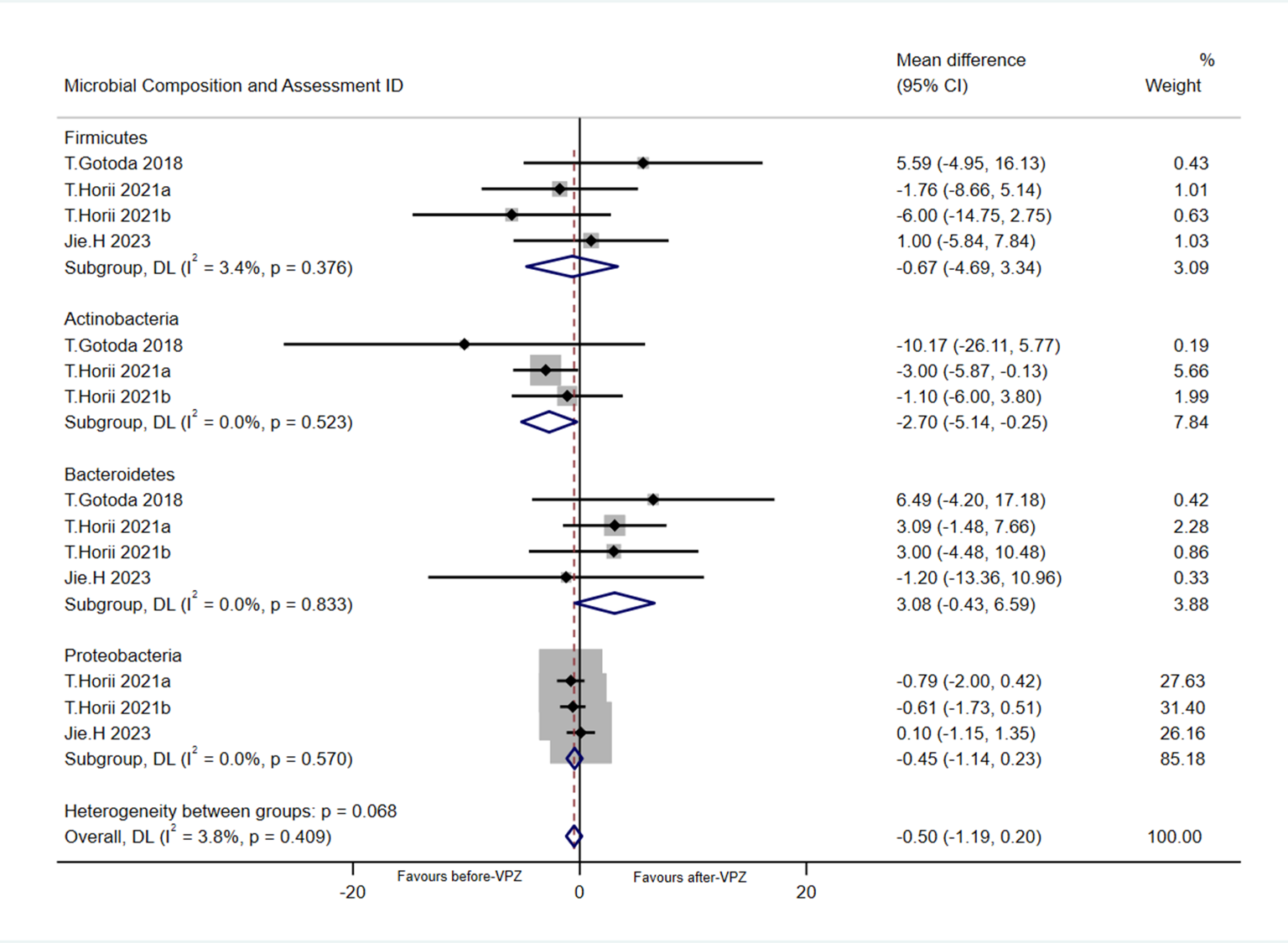
**

**Supplementary Figure 3.** Forest plots of changes in gut microbiota at the phylum level 1-3 months after VPZ treatment.

**Supplementary Figure 4.
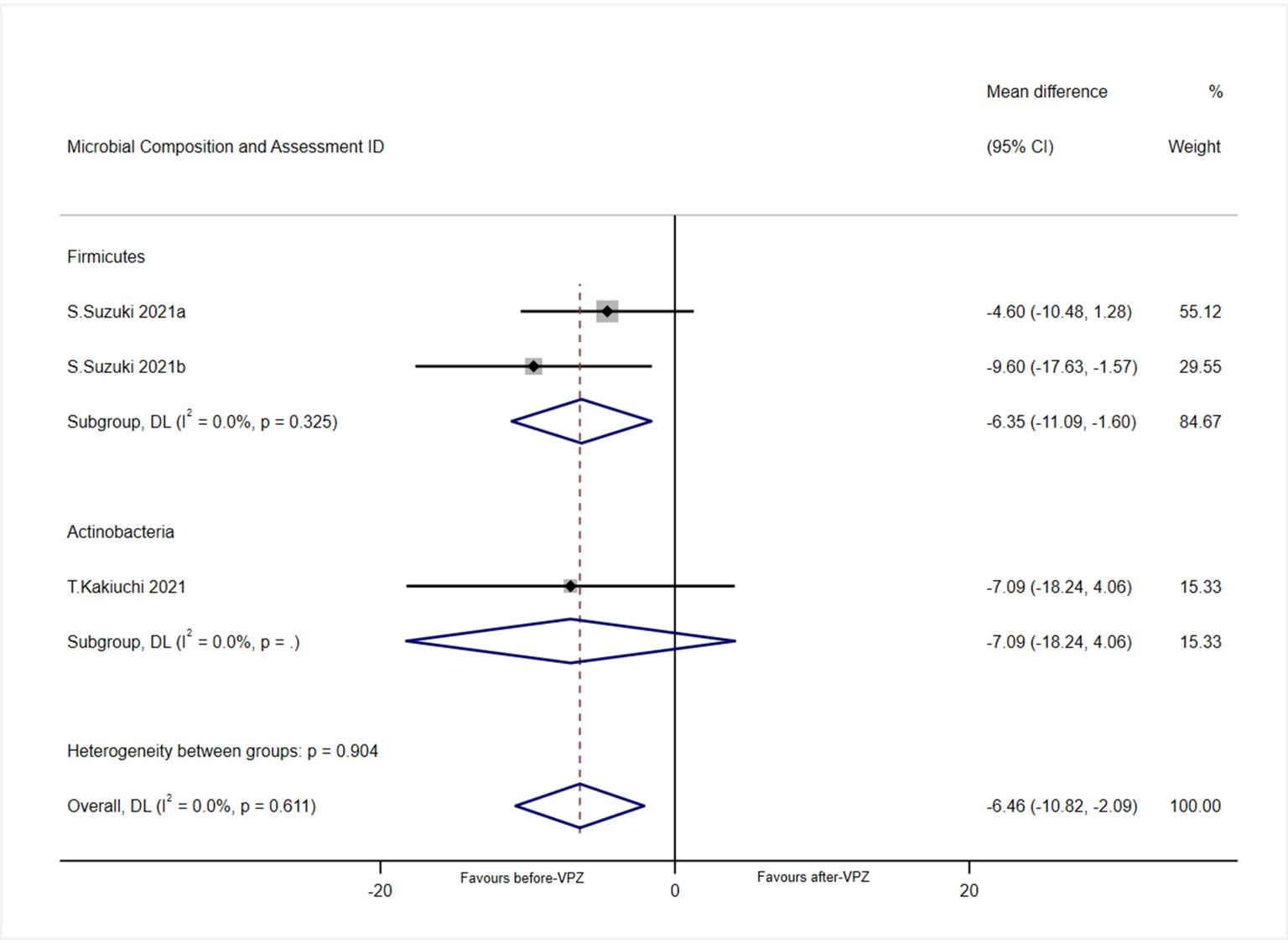
**

**Supplementary Figure 4.** Forest plots of changes in gut microbiota at the phylum level over 3 months after VPZ treatment.

**Supplementary Figure 5.
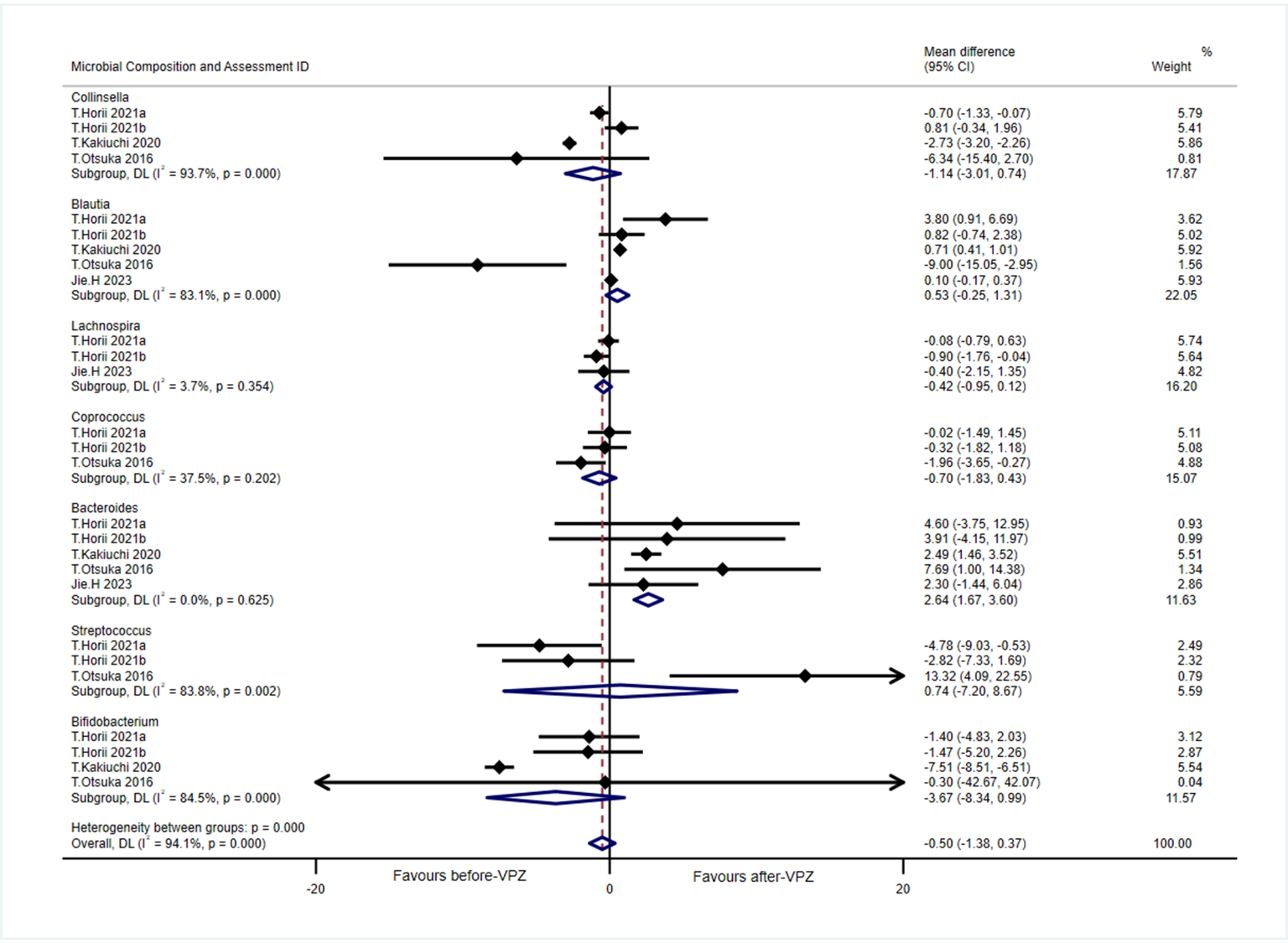
**

**Supplementary Figure 5.** Forest plots of changes in gut microbiota at the genus level 1-3 months after VPZ treatment.
